# Results of bacterial cultivation are infrequently utilized in the treatment of patients hospitalized with severe odontogenic infections – a retrospective cohort study

**DOI:** 10.1080/20002297.2025.2603683

**Published:** 2025-12-19

**Authors:** Rasmus Søndenbroe, Merete Markvart, Daniel Belstrøm, Frederik Boëtius Hertz, Thomas Bjarnsholt, Claus Henrik Nielsen, Sanne Werner Møller Andersen, Simon Storgård Jensen

**Affiliations:** a Department of Odontology, Section of Oral Biology and Immunopathology, University of Copenhagen, Copenhagen N, Denmark; b Department of Odontology, Section of Clinical Oral Microbiology, University of Copenhagen, Copenhagen N, Denmark; c Department of Clinical Microbiology, Copenhagen University Hospital, Copenhagen N, Denmark; d Department of Immunology and Microbiology, University of Copenhagen, Copenhagen N, Denmark; e Department of Oral and Maxillofacial Surgery, Copenhagen University Hospital, Copenhagen N, Denmark

**Keywords:** Mouth diseases/microbiology, drug resistance, anti-bacterial agents/pharmacology, anti-bacterial agents/therapeutic use, tooth diseases/drug therapy, bacterial infections/diagnosis, tooth diseases/microbiology

## Abstract

**Background:**

Patients hospitalized with severe odontogenic infections (SOI) receive empiric intravenous antibiotics. Microbiological cultivation and antibiotic susceptibility testing are commonly performed, although the clinical value is debated.

**Objective:**

To assess the value of routine microbiological cultivation and susceptibility testing in patients hospitalized with SOI.

**Design:**

This retrospective cohort study included patients hospitalized with SOI, at the University Hospital of Copenhagen, Denmark, from November 2012 to 2019. Data on microbiological cultivation, bacterial identification and antibiotic susceptibility testing were obtained from hospital records. Statistical analysis included χ² test, Fisher's exact test, analysis of variance and logistic regression.

**Results:**

A total of 384 patients were included, with microbiological data available for 243 patients. Antibiotic treatment was modified in 47 patients and in seven cases, the modification was based on cultivation and antibiotic susceptibility testing. Higher age was associated with the need for cultivation and susceptibility testing (*p* = 0.006). The infections were polymicrobial, predominantly involving resident oral microbiota. *Streptococcus* was the most frequent genus (34% of isolates). Penicillin resistance was observed in 30% of all isolates.

**Conclusion:**

Testing rarely influences antibiotic management in SOI. Higher age showed limited predictive value. The high prevalence of penicillin resistance among patients with SOI warrants further investigation.

## Introduction

Infections with an odontogenic origin can exacerbate, become purulent and develop into an abscess. The odontogenic abscess can enter into a more severe state, penetrate the alveolar bone, spread into adjacent anatomical spaces and have severe life-threatening consequences, which require treatment in a hospital setting [1–6]. Severe odontogenic infections (SOI) are in different studies, defined on various diagnostic definitions, but are generally defined as an infection in the head and neck, with odontogenic origin, that requires hospital admission and specialist treatment [4–11].

SOI is a global health issue, with an annual incidence between 7.2 and 74.3 out of 100,000 inhabitants, and are increasing both in incidence and severity [6,11–15]. Patients hospitalised with SOI are, in most cases, treated with broad-spectrum intravenous antibiotics [16–18]. Empiric intravenous antibiotic treatment is initiated upon hospital admission to facilitate timely intervention and minimise the spread of infection. The majority of patients hospitalised with SOI undergo surgery, during which routine microbiologic sampling is performed for cultivation and identification of antibiotic susceptibility [17].

Studies using both cultivation and sequencing have shown that SOI are polymicrobial infections [16,19–24]. The microbiology of the SOI consists primarily of both anaerobic and aerobic bacteria, and species that are a resident part of the oral microbiota [20,22,23,25–30]. The search for a culprit bacterium, a single one, or even a specific group of pathogens to be considered the major pathogen involved with exacerbation of odontogenic infections has proven fruitless [22,23].

Routine cultivation and susceptibility testing rarely influence treatment, since most patients (92–94%) are discharged before the results are available [16,31]. With the increasing need for optimising healthcare resource allocation, the necessity of systematic cultivation and antibiotic susceptibility testing in SOI patients warrants critical evaluation [16,31]. Cultivation and antibiotic susceptibility testing have been proposed to be limited to high-risk groups, including immunocompromised individuals, patients in intensive care (ICU), those with postoperative complications and those requiring multiple surgical interventions [16,31]. The key measure of value is the number of patients who must be tested before one patient's treatment changes, also known as the number needed to test. To stratify patients who need cultivation and susceptibility testing, the number needed to test is essential. Furthermore, an in-depth analysis of the cause of treatment, the microbiological composition and potential indicators for modifying antibiotic treatment is needed.

We hypothesise that the clinical impact of cultivation and susceptibility testing in the management of SOI is minimal, as the results are infrequently incorporated into clinical decision-making. In this study, we aimed to determine the numbers needed to test for systematic cultivation and antibiotic susceptibility testing in patients hospitalised with SOI. Furthermore, we aim to define predictive patient-related factors for the need for cultivation and antibiotic susceptibility testing.

## Method

### Study design/setting

In this present retrospective cohort study, we included patients hospitalised with SOI at the Department of Oral and Maxillofacial Surgery, Copenhagen University Hospital, Denmark, from 1 November 2012, to 31 December 2019. The study was approved by the Danish Patient Safety Authority (registration: 3-3013-2349/1) and the Capital Region of Denmark Centre of Data (registration: *P*-2019-841).

### Participants

We collected data on potentially eligible patients through the electronic medical record system (Electronic Health Records, EPIC). The search was performed among all patients hospitalised with an abscess or phlegmone in the oral cavity (WHO ICD-10 diagnosis K12.2 and Danish version DK12.2). Patients were included if the following four criteria were met: i) diagnosis, abscess or phlegmone in the oral cavity, ii) odontogenic origin of the infection, iii) the patient had been hospitalised at least one night and iv) sufficient medical records were available. We excluded the patients if: i) the infection did not have an odontogenic origin, ii) medical records were missing.

### Data

In accordance with our aims and hypotheses, we defined the outcome variables as bacterial species taxonomic classification, antibiotic susceptibility, modification of antibiotic treatment based on microbiological test results, and the number needed to test. The outcome variable, ‘*modification of antibiotics based on microbiological test results’,* was defined according to the reason documented by the maxillofacial surgeon in the hospital records for altering the antibiotic prescription.

Potential predictive factors were identified. These potential predictive factors were Age (continuous variable in whole years), Antibiotic prior to admission (yes/no), Diabetes mellitus (yes/no), hypertension (yes/no), psychiatric disorders (yes/no), chronic obstructive pulmonary disease (yes/no) and asthma (yes/no). We defined potential confounders as sex/gender (male/female), Alcohol consumption (yes/no), Smoking (yes/no), paraclinical test results (C-reactive protein (CRP) at first day of hospitalisation (continous variable mg/L), leucocyte counts at first day of hospitalisation (continous varaible 10⁹/L) and the odontogenic origin of the infection; apical periodontitis post extraction infection, pericoronitis, multiple foci, unknown and other.

All data were extracted from hospital medical records by a single investigator (RS), with consultation from a second investigator (SWMA) in cases of uncertainty. To determine the origin of infection, we used a combination of hospital records, radiographic analysis and treatment records. In cases of uncertainty of the origin of infection, this variable was categorised as ‘unknown’.

### Microbiological data

From the hospital records, we included results from microbial culturing and antibiotic susceptibility testing of pus from the abscesses. The taxonomic classification of bacterial species had been determined using Matrix-assisted laser desorption ionisation-time of flight mass spectrometry (MALDI-TOF MS) (Bruker Daltonics, Bremen, Germany), in accordance with the standard procedure applicable at the time of the study period [32]. Antibiotic susceptibility testing was performed using the Agar diffusion method, and an antibiogram was generated. Per 2 November. In 2015, implementation of the guideline from the European Committee on Antimicrobial Susceptibility Testing (EUCAST) 2024 [33] was implemented at the Copenhagen University Hospital Department of Clinical Microbiology. Antibiotic resistance prior to 2 November 2015, was cultivated on Danish Blood Agar plates and Rosco tablets, resulting in semi-confluent growth. After 2 November 2015, standardised inoculum 0.5 McFarland was introduced, resulting in confluent growth. The cultivation results were divided into atmospheric growth conditions (aerobic, anaerobic and facultative anaerobic), and Gram-staining characteristics were determined by the principal investigator (RS). The media used for resistance testing were Mueller‒Hinton fastidious broth and Oxoid disks. Data prior to 2 November 2015, were reported with the numeric values 3-2-1-0, and after Susceptible (S), Intermediate (I) and Resistant (R), the conversions were 3 = S, 2 = I, 1 and 0 = R.

The results of antibiotic susceptibility testing were reported following the classification of the Antibiotic susceptibility testing was performed depending on species found and included the following antibiotics: penicillin (penicillin, ampicillin, amoxicillin + clavulanic acid, dicloxacillin, methicillin and piperacillin + tazobactam), cephalosporins (ceftazidim, ceftriaxon, cefuroxim), macrolides (azithromycin, erythromycin), quinolones (ciprofloxacin, moxifloxacin), aminoglycosides (gentamicin, tobramycin) and other (aztreonam, chloramphenicol, clindamycin, colistin, linezolid, meropenem metronidazole, rifampicin, sulfonamid, trimethoprim, trimethoprim + sulfamethoxazol and vancomycin). Antifungals tested were amphotericin, anidulafungin, caspofungin, flucunazol, fucidin, micafungin and voriconazol.

Study data were collected and managed using REDCap (Research Electronic Data Capture) tools hosted at Copenhagen University Hospital [34,35].

For the calculation of numbers needed to test, the total number of tests was divided by the number of patients for whom the treatment with intravenous antibiotics was modified based on cultivation and antibiotic susceptibility testing.

### Statistical analysis

We conducted a power calculation with 80% power and an alpha level of 0.05. Based on prior data showing that microbiological samples were cultivated in 23% of hospitalised patients, and 4.3% of patients were changed in antibiotic therapy [16]. This power calculation resulted in two required sample sizes: a minimum of 320 patients for the total study cohort, and a minimum of 127 patients with cultivated microbiological samples for the subgroup analyses.

In the analysis, we applied age as a continuous variable, despite calculating age in whole years. We applied CRP and leucocyte counts as continuous variables with one decimal. We analysed statistical differences in the distribution of potential predictor variables in outcome variables using the Pearson χ² or Fisher's exact test on categorical variables. Analysis of variance (ANOVA) was used to analyse the distribution of continuous variables between the different groups of the outcome variables. Shapiro–Wilk test was applied to test the normal distribution of these variables, and the Kruskal‒Wallis test was used for non-parametric testing. We tested for multicollinearity with the use of variance inflation factors. We fitted a multivariable logistic regression model to determine the effect of predictors, controlled for confounders and effect modifiers. The overall adequacy of the models was tested by the Omnibus Tests of Model Coefficients and the Hosmer and Lemeshow goodness of fit test. The capability of the models to explain variability in data was tested according to Nagelkerke *R2*. To control for the potential effect of missing data, we assessed this for all covariates after modelling. All descriptive and analytic statistics were performed in RStudio version 2023.12.0.369, using the packages dplyr, ggplot2, scales and tidyr and in Statistical Package for the Social Sciences (SPSS) version 28.0.0.0. SPSS. The significance level in all tests was set at *p* < 0.05. Table 1.

**Table 1.** Patient characteristics stratified by reason for modification of antibiotic treatment during hospitalisation.

Variable	Microbiological results (*n* = 7)		Other cause (*n* = 40)		No modification (*n* = 196)		*p*-value
Age, years mean (±SD)	68.0	(±16.1)	49.6	(±17.6)	43.92	(±19.3)	0.002*
Sex/gender, *n* (%)							
Female	5	(71.4)	14	(35.0)	92	(46.9)	0.147
Male	2	(28.6)	26	(65.0)	104	(53.1)	
Alcohol consumption							
Yes	3	(42.9)	27	(67.5)	91	(46.4)	0.038*
No	1	(14.3)	4	(10.0)	52	(26.5)	
Missing	3	(42.9)	9	(22.5)	53	(27.0)	
Smoking							
Yes	0	(0.0)	17	(42.5)	69	(35.2)	0.061
No	5	(71.4)	13	(32.5)	80	(40.8)	
Missing	2	(28.6)	10	(25.0)	47	(24.0)	
CRP first day mean, mg/L (±SD)	252.3	(±169.6)	170.9	(±125.2)	149.2	(±108.8)	0.047*
Leucocytes mean, 10⁹/L (±SD)	17.1	(±5.3)	15.8	(±5.7)	14.37	(±5.4)	0.185
Diabetes mellitus	2	(28.6)	6	(15.0)	13	(6.6)	0.036*
Hypertension	3	(42.9)	12	(30.0)	18	(9.2)	<0.001*
COPD	2	(28.6)	4	(10.0)	12	(6.1)	0.071
Asthma	1	(14.3)	2	(5.0)	10	(5.1)	0.580
Psychiatric disorder	1	(14.3)	5	(12.5)	30	(15.3)	0.901
Origin of infection							
Apical periodontitis	1	(14.3)	15	(37.5)	94	(48.0)	0.131
Post extraction infection	3	(42.9)	9	(22.5)	54	(27.6)	
Pericoronitis	0	(0.0)	3	(7.5)	12	(6.1)	
Other	2	(28.6)	4	(10.0)	14	(7.1)	
Unknown	1	(14.3)	2	(5.0)	4	(2.0)	
Multiple Foci	0	(0.0)	7	(17.5)	18	(9.2)	
Antibiotic prior to admission							
Yes	4	(57.1)	18	(45.0)	136	(69.4)	0.009*
No	3	(42.9)	22	(55.0)	58	(29.6)	

Abbreviations: Standard Deviation (SD), C-reactive protein (CRP), Chronic Obstructive Pulmonary Disease (COPD).

## Results

**Figure 1.**
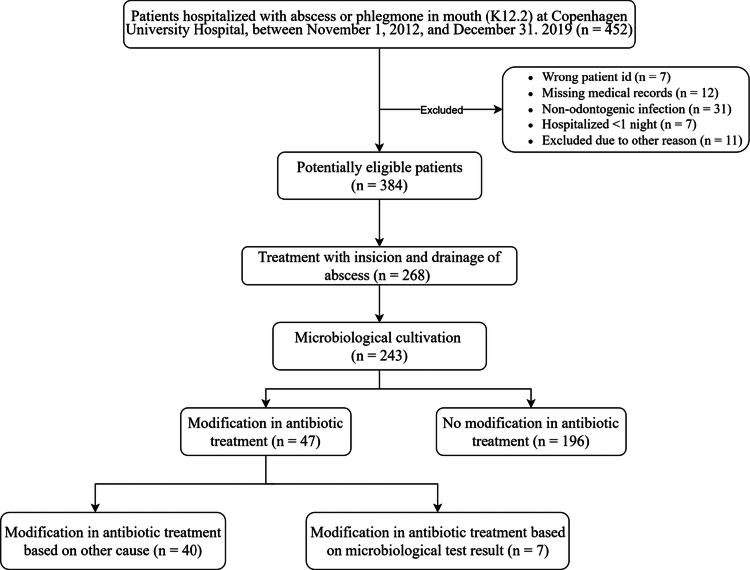
Selection process of patients included in this study.

A total of 384 unique patients hospitalised with SOI were potentially eligible, of whom microbiological cultivation was performed in 243 cases (60.6%). The inclusion and exclusion processes are displayed in Figure 1. The data included in this study exceeded both sample size requirements determined by our power calculation (required: 320 total patients and 127 cultivated samples; achieved: 384 patients and 243 cultivated samples).

Antibiotic modification was performed on average one day after hospital admission. The modification followed microbiological test results, corresponding to 2.9% of all patients hospitalised with SOI (Table 2). From these results, the number needed to test was ⌈34.71⌉ = 35.

**Table 2.** Frequency table of the modification of antibiotic therapy.

	*n*	(%)
Incorrect prescription	23	(48.9)
Suspected necrotising soft tissue infection	8	(17.0)
Microbiological test result	7	(14.9)
No clinical improvement	4	(8.5)
Unknown	3	(6.4)
Allergic reaction	1	(2.1)
No improvement in paraclinical parameters	1	(2.1)
Total	47	(100.0)

Note: Incorrect prescriptions were deviations from department protocol, e.g. penicillin and amoxicillin.

The empirical prescription of antibiotics according to the guidelines of the Department of Oral and Maxillofacial Surgery was intravenous (IV) cefuroxime 1,500mg in combination with IV metronidazole 500mg three times daily. Antibiotic therapy was modified during hospitalisation in 47 patients. Equivalent to 20.1% of patients with underwent microbiological testing, and 12.2% of the entire cohort. In most cases, the antibiotic modification was due to incorrect prescription (Table 2). Typically, the patient was initially given penicillin in combination with metronidazole at the emergency room and was modified to the empiric prescription at the inpatient ward according to the existing guidelines.

### Microbiological test results

**Figure 2.**
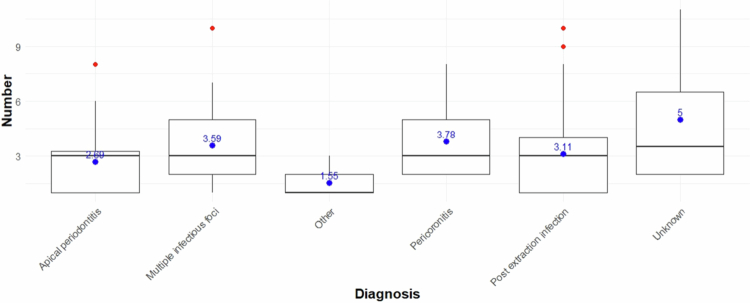
Distribution of the number of isolates by origin of infection. Mean values (blue). Outliers (red). Significant differences were found between the ‘Other’ group and both ‘Multiple infectious foci’ and ‘Pericoronitis’ (Kruskal–Wallis χ² = 13.53, df=5, *p* = 0.019; Wilcoxon post hoc tests with Bonferroni-adjusted *p*-values).

We found that 27.2% of samples had one isolate, 22.0% had two, 24.3% had three and 26.5% had >4, indicating that most samples were multispecies (72.8%). The mean number of isolates was 2.9. The number of isolates divided by clinical diagnosis group is displayed in Figure 2. Our pairwise comparison of origin of infection showed significant differences between the group ‘Other’ and the groups ‘Multiple infectious foci’ and ‘Pericoronitis’ (Kruskal–Wallis chi-squared = 13.532 with degrees of freedom of 5, and a *p*-value = 0.01887). These pairwise comparisons were made using the Wilcoxon rank sum test with continuity correction. The *p*-values were adjusted using Bonferroni correction (Figure 2).

**Figure 3.**
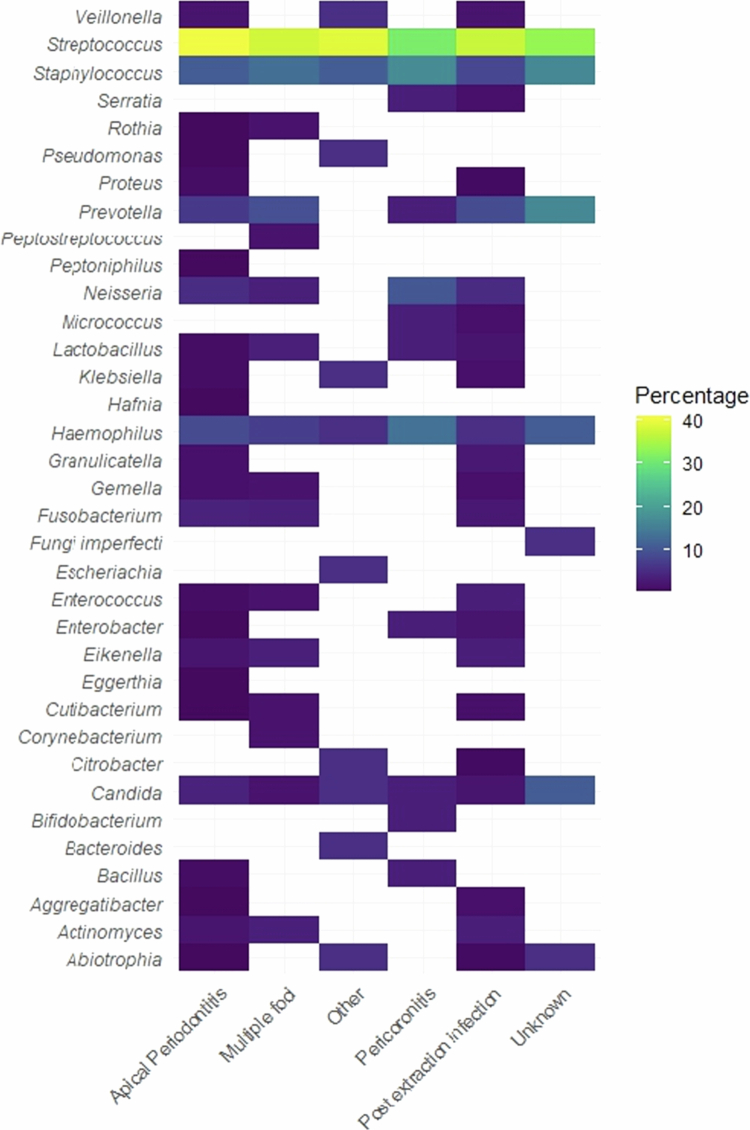
Prevalence of bacterial genera in microbiological test results.

**Figure 4.**
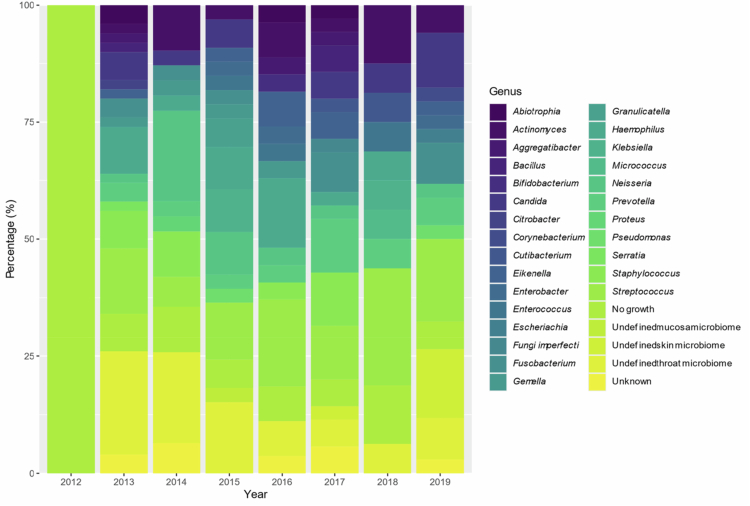
Annual distribution of cultivation results by genera.

We identified 35 genera and 83 species across 353 samples. *Streptococcus* was the predominant genus, identified in 34.0% of all isolates (Figure 3). Other genera frequently identified (all >5%) were *Staphylococcus* (10.6%), *Prevotella* (8.5%), *Haemophilus* (7.4%) and *Neisseria* (6.2%) (Figure 3). Results of cultivation over the study period are displayed in Figure 4. The most predominant species were *Streptococcus anginosus (13%), Staphylococcus epidermidis* (8%), *Haemophilus parainfluenzae* (7%), *Streptococcus mitis* (6%) and *Prevotella buccae* (6%) (Supplementary Figure S1)*.* Due to the wide distribution of genera and species, resulting in few observations in each category, no statistical tests were performed to assess the distribution of species or genera by origin of infection. We tested the distributions of atmospheric growth conditions and Gram staining by origin of infection. After excluding non-relevant and unknown atmosphere categories, we found no significant association with the use of Pearson χ² or Fisher's exact test (*p* = 0.23 and *p* = 0.65).

Due to changes in the culturing procedure during the study period, we performed a temporal analysis of the cultivation results. We detected no significant change in the temporal analysis of cultivation results. Before November 2015, 152 samples (81.3%) were classified at the genus or species level. After November 2015, 169 samples (85.8%) were classified at the genus or species level. There was no significant difference in the distribution of these classifications before and after November 2015, when a new cultivation procedure was introduced (χ² = 1.11, *p* = 0.292). A fitted logistic regression model of the annual proportion of samples classified to the genus or species level indicated an increase over time (OR per year=1.06), but we did not find a significant temporal trend (95% CI 0.92–1.21, *p* = 0.432) (Figure S2).

### Antibiotic susceptibility testing

**Figure 5.**
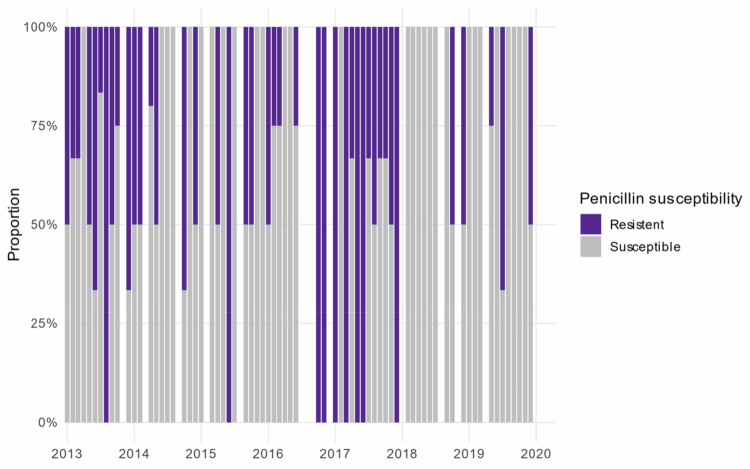
Monthly proportion of penicillin-resistant and penicillin-susceptible isolates.

**Figure 6.**
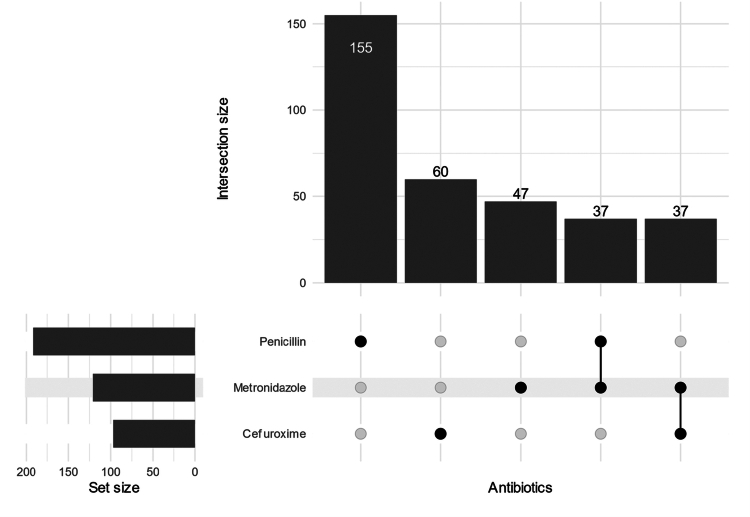
UpSet plot of prevalence of antibiotic resistance.

We found that of all the cultivated bacteria, 155 (30.0%) displayed phenotypic resistance to penicillin (Figure 5). Out of these penicillin-resistant bacteria, 9.0% would be theoretically susceptible to metronidazole. The susceptibility test showed that 10 (1.9%) of all isolates displayed phenotypic resistance to metronidazole. The susceptibility test revealed that 37 (23.9%) of the penicillin-resistant isolates exhibited resistance to cefuroxime, accounting for 7.2% of all isolates (Figure 6). According to susceptibility testing results, 37 (23.9%) showed resistance to the empiric antibiotic combination of metronidazole and cefuroxime, the same number as with penicillin and metronidazole (Figure 6). The mean number of isolates did not differ substantially between penicillin-resistant (4.28) and penicillin-susceptible (4.11) isolates, indicating no clear relationship between sample polymicrobiality and penicillin resistance.

We used a logistic regression model to assess whether penicillin resistance changed over time. The analysis showed no significant temporal trend in resistance (OR = 1.00, 95% CI: 0.999–1.00, *p* = 0.46), indicating that the proportion of penicillin-resistant isolates remained stable throughout the study period. The temporal distribution of penicillin resistance is shown in Figure 5, excluding the 2012 results because only one sample was available.

### Modification of antibiotic therapy

The bivariate analysis showed that age, alcohol consumption, CRP at the first day of hospitalisation, diabetes mellitus, hypertension and antibiotics prior to admission were significantly associated with the modification of antibiotic therapy based on cultivation and antibiotic susceptibility testing (*p*<0.05) (Table 1). Variance inflation factors for these variables ranged from 1.01 to 1.33, indicating no multicollinearity. These variables were included in our first multiple logistic regression model (Table 3). To assess the robustness of our findings, we conducted a sensitivity analysis excluding covariates with >20% missing data (alcohol consumption and smoking). The reduced logistic regression model yielded estimates that were consistent in direction and magnitude with the primary complete-case model. Overall, the sensitivity analysis supported that missing data did not materially influence the conclusions.

**Table 3.** First multiple logistic regression with predictive factors of patients in need of cultivation and susceptibility testing.

		Outcome			
*Predictors*	*Odds ratio*	*CI*			*p*
Age	1.110	0.986	–	1.249	0.084
Alcohol consumption	1.007	0.998	–	1.016	0.129
CRP first day	1.779	0.114	–	27.721	0.681
Diabetes mellitus	9.431	0.776	–	114.67	0.078
Hypertension	0.610	0.043	–	8.6924	0.715
Antibiotic prior to admission	0.744	0.074	–	7.4538	0.802
(Intercept)	0.000				

Abbreviations: CRP: C-reactive protein, * significant. Omnibus Tests of Model Coefficients = 14.12, *p* = 0.028, Hosmer–Lemeshow = 1.05, *p* = 0.998.

The first multivariate model revealed that none of the included predictors or confounders showed a significant association with modification of antibiotic therapy based on cultivation and antibiotic susceptibility testing (Table 3). We subsequently conducted a model search with backward elimination. We excluded variables one at a time, starting with the variable with the highest *p*-value, and we then refitted the model. Variables were excluded in the following order: antibiotic use prior to admission, alcohol consumption, hypertension, diabetes mellitus and CRP at first day. In the final model, age was significantly associated with a modification of antibiotic therapy based on cultivation and antibiotic susceptibility testing (OR = 1.071, CI=1.020–1.125, *p* = 0.006), corresponding to a 7% increase in odds per additional year (Table 4). The final logistic regression model was able to predict between 4.0 and 17.5% of the variance in the outcome (Cox & Snell R² = 0.04; Nagelkerke R² = 0.175) (Table 4).

**Table 4.** Final multiple logistic regression with predictive factors of patients in need of cultivation and susceptibility testing.

		Outcome			
*Predictors*	*Odds ratio*	*CI*			*p*
Age	1.071	1.020	–	1.1248	0.006*
(Intercept)	0.001				

Abbreviations: * significant. Omnibus tests of model coefficients = 9.999, *p* = 0.002, Hosmer–Lemeshow = 4.979, *p* = 0.760.

## Discussion

Our study showed that systematic cultivation and susceptibility testing rarely led to the modification of antibiotic therapy in patients suffering from SOI. In our analysis, for every 35 cultivations performed, only one resulted in a modification of the antibiotic regimen for patients with SOI. This is a confirmation of our hypothesis that the clinical impact of systematic cultivation and susceptibility testing in the management of SOI is minimal, as results are infrequently incorporated into clinical decision-making, which aligns with previous findings [16,31]. However, this relatively low adjustment rate should not be interpreted solely as a lack of utility. The antibiotic treatment of SOI varies [16,18,31,36]. In our study, the empiric regimen consisted of cefuroxime combined with metronidazole, providing broad-spectrum coverage against the vast majority of pathogens identified. As a result, most cultivation results would confirm the adequacy of empiric therapy rather than prompting a modification. A narrower empiric antibiotic treatment might enhance the clinical relevance of systematic cultivation. Moreover, the majority of the patients in this cohort were discharged after three days [17], which is before cultivation results are complete, confirming similar findings and indicating the redundancy of systematic cultivation [16,31]. In some cases, cultivation results can be necessary for acute life-saving treatment and clinical decision-making [16]. The development of SOI into cervical necrotising fasciitis or into other cases in need of intensive care can be fatal. Therefore, in acute life-threatening cases or when resistance patterns are suspected, cultivation remains indispensable regardless of the observed. Furthermore, to support local, regional and global surveillance of resistance patterns in oral infections, cultivation of SOI can be a valuable source of data. Therefore, maxillofacial clinics should consider both the clinical and the theoretical importance of systematic cultivation and susceptibility testing.

Our analysis did not identify reliable predictors for when cultivation would lead to modification of antibiotic therapy. Previously, other reports have suggested that systematic cultivation is unnecessary in patients with single-space infections [16,31]. In this study, we were unable to test this hypothesis due to insufficiently reliable data.

Although we have shown that several variables – including age, CRP at admission, alcohol consumption, hypertension, diabetes mellitus and prior antibiotic exposure – were significantly associated with the outcome in univariate analyses, only age remained associated in the multivariate model. The model explained only 4–18% of the variance, indicating limited clinical usefulness. Sparse data, including a very small number of patients with treatment modification, likely contributed to wide confidence intervals and model instability. The effect size for age (OR 1.071), although statistically significant, is small and unlikely to be of clinical relevance and this finding should be interpreted with caution.

The microbiota of SOI are generally polymicrobial and consist primarily of bacteria that are constituents of the resident oral microbiota [16,20–23]. The number of different species we found is similar to earlier findings [20]. The majority of the samples were polymicrobial, in accordance with previously reported [16,19–23,27]. Our study extends this knowledge by indicating that higher microbiological diversity was associated with apical periodontitis, multiple infectious foci and unknown infectious foci as the origins of infection.

*Streptococcus* was the most frequently identified genus, in agreement with previous literature [16,20,25,26]. The predominance may partly reflect the ease of cultivation of *Streptococcus* in standard culture-based methods [23]. In contrast, *Prevotella*, *Porphyromonas*, *Fusobacterium* and *Parvimonas* may be insufficiently detected by current culture-based methods [23]. We identified *Prevotella* and *Fusobacterium* at 8.5 and 2.5%, respectively, consistent with other findings [25]. *Streptococcus anginosus,* the most prevalent species in our study, has previously been found in 28.7 and 49% of microbiological samples from SOI [26,37]. Odontogenic infections comprised of bacteria from the *Streptococcus anginous* group have been shown to be more complicated, severe and life-threatening [26]. Due to the low level of ICU admissions, we were unable to test this in this study. *Prevotella* species have been suggested to contribute to the development, exacerbation and spreading of an odontogenic infection into a severe, life-threatening state [38]. In this study, we were unable to identify a specific bacterium as the culprit, and we did not detect any differences in microbiological profile depending on the origin of infection. Therefore, preventive strategies aligned with antibiotic stewardship should consider these patient groups as having similar causative bacteria when aiming to reduce the incidence of SOI. Furthermore, given the similarity between the microbiota of SOI and that reported in cerebral abscesses of oral origin [2], there is an underlying need for prospective studies that investigate risk factors for the spread of commensal oral bacteria.

The proportion of penicillin-resistant bacteria in these samples where surprisingly high, but aligns with findings from other studies [2,5,27] and is higher than the prevalence reported elsewhere [39]. A recent study of saliva samples from Danish adults showed that 7.8% displayed resistance to penicillin [40]. Furthermore, in odontogenic abscesses, an increase in the prevalence of *β*-lactam-resistant microorganisms over the period of 1991–2005 was reported [36]. These findings of a high prevalence of antibiotic resistance in the resident oral microbiota reinforce the focus on avoiding unnecessary antibiotic use and the need for antibiotic stewardship in odontology. Furthermore, from a clinical perspective, the observed rate of penicillin resistance underscores the importance of patient follow-up after antibiotic prescription in primary dental care, given the potential risk of treatment failure due to resistance. Furthermore, our results suggest that penicillin monotherapy may be insufficient in a substantial proportion of patients with SOI. When considering antibiotic treatment for patients at risk of exacerbation of an odontogenic infection, it should be discussed whether monotherapy with penicillin is adequate or whether combination therapy with penicillin and metronidazole is preferable. The logistic regression analysis did not reveal evidence of a modification of penicillin resistance over time.

Our study has several limitations due to its retrospective design, including missing data, variation in cultivation procedures over time and possible misclassification of diagnoses. Although we did not identify systematic temporal changes in antibiotic susceptibility or microbiological findings, inconsistent sampling methods may have introduced bias. Despite meeting sample size requirements from our power calculation, the low number of antibiotic modifications limits the robustness of the regression model. A diverse composition of the microbiology of odontogenic infections has been reported globally [41]. This can be due to different microbial identification techniques [41].

Future prospective studies using standardised cultivation protocols and comprehensive data collection are needed to validate the microbiological profile of SOI, assess the prevalence of antibiotic resistance and define which patients benefit most from systematic microbiological analysis.

## Conclusion

This study confirms that the results of systematic cultivation and antibiotic susceptibility testing are of limited clinical value. Only age showed a significant effect on the need for cultivation and susceptibility testing, but due to statistical limitations, this result should be interpreted with caution. Although the clinical relevance of systematic microbiological testing appears limited, it nevertheless has theoretical value. Since 30% of all samples from these infections showed penicillin resistance, systematic cultivation can be essential for antibiotic susceptibility surveillance and antibiotic stewardship.

## Supplementary Material

Supplementary material Figure S1 and S2
