# Supplementary material for: Results of bacterial cultivation are infrequently utilized in the treatment of patients hospitalized with severe odontogenic infections – a retrospective cohort study
Source: J Oral Microbiol. 2025 Dec 19;17(1):2603683. doi: 10.1080/20002297.2025.2603683 (PMC12720675; doi:10.1080/20002297.2025.2603683)
Supplement: Supplementary material — Figure S1 and S2 [file ZJOM_A_2603683_SM8307.docx]

# Supplementary


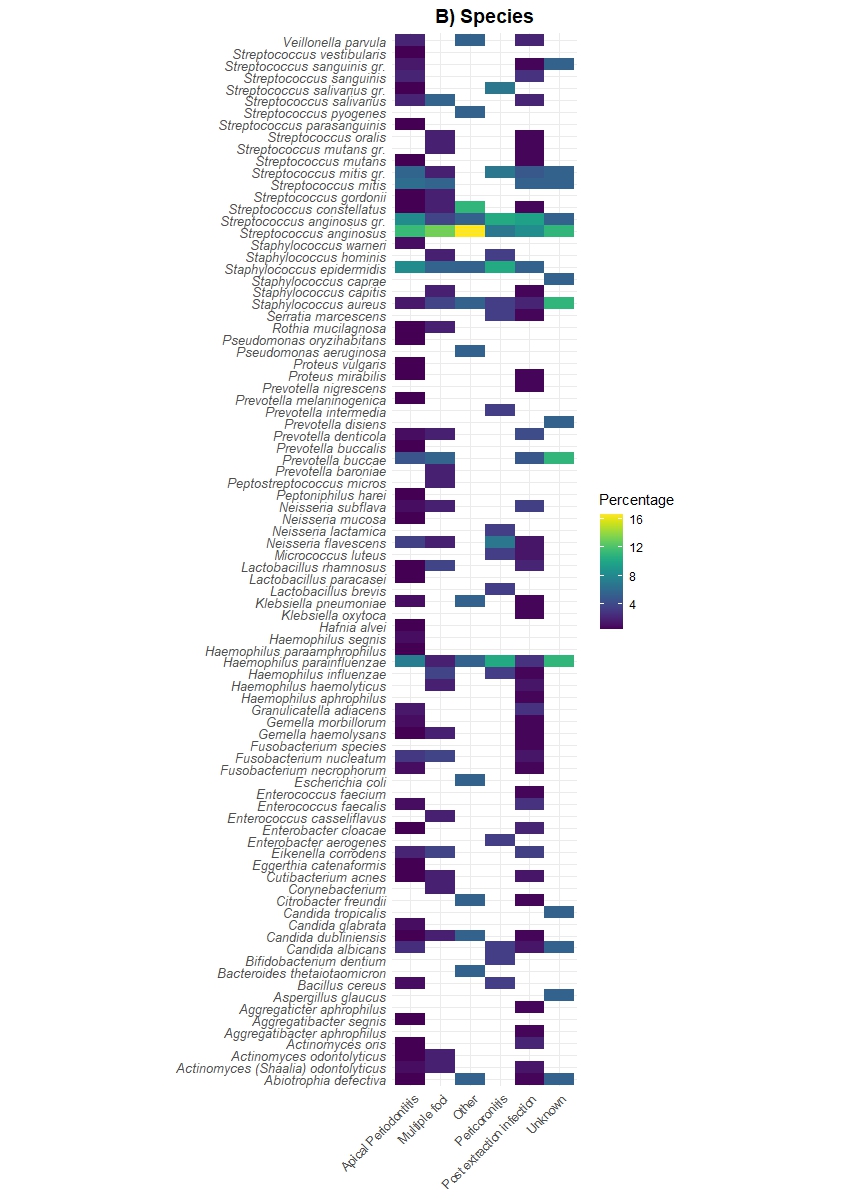
Figure S1 - Prevalence of bacterial species in microbiological test results

Figure S2 – Prevalence of microbiological test results of genera og species, year 2013-219. Including a fitted logistic regression model.


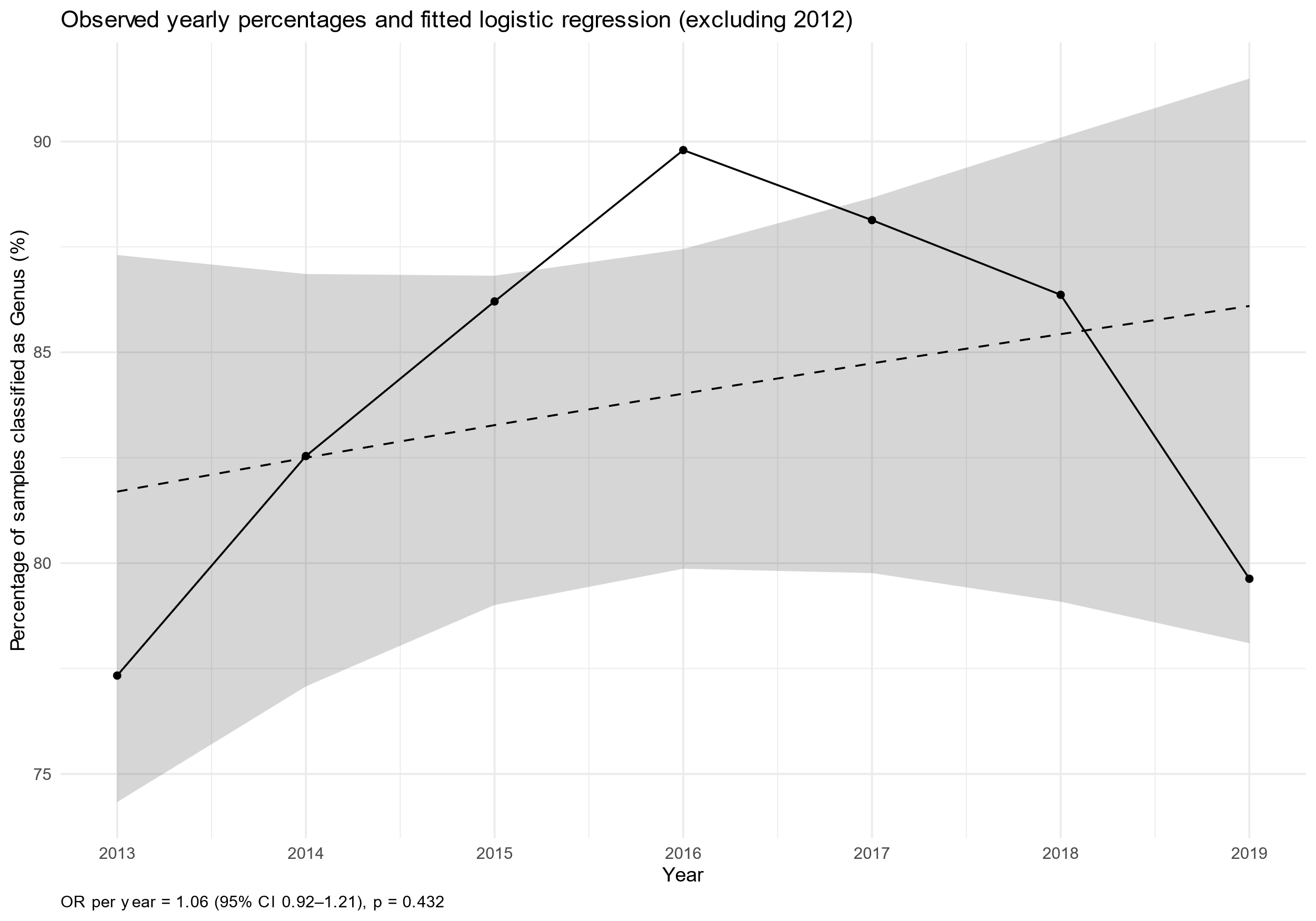


Odd ratio per year =1.06 (95 % CI 0.92-1.21), p = 0.432.
